# Investigating Genetic and Phenotypic Variability of Queen Bees: Morphological and Reproductive Traits

**DOI:** 10.3390/ani11113054

**Published:** 2021-10-26

**Authors:** Elena Facchini, Maria Grazia De Iorio, Federica Turri, Flavia Pizzi, Daniela Laurino, Marco Porporato, Rita Rizzi, Giulio Pagnacco

**Affiliations:** 1 Department of Veterinary Medicine, Università degli Studi di Milano, Via Celoria 10, 20133 Milan, Italy; elena.balla@gmail.com (E.F.); rita.rizzi@unimi.it (R.R.); giulio.pagnacco@unimi.it (G.P.); 2 Institute of Agricultural Biology and Biotechnology, National Research Council, Via Einstein, 26900 Lodi, Italy; federica.turri@ibba.cnr.it (F.T.); flavia.pizzi@ibba.cnr.it (F.P.); 3 Department of Agricoltural, Forest and Food Sciences, University of Turin, Largo Paolo Braccini 2, 10095 Grugliasco, Italy; daniela.laurino@unito.it (D.L.); marco.porporato@unito.it (M.P.)

**Keywords:** honeybee queen, bee breeding, morphological traits, reproductive traits, heritability, genetic parameters

## Abstract

**Simple Summary:**

Honeybees have attracted considerable scientific and public interest in recent years. Besides pesticides and pathogens, failure or loss of the queen have been considered the most important factors leading to colony losses worldwide. The poor quality of the queen is a factor that ranks among the top reasons for bee colony failure. There are traits that can indicate the quality of a queen bee. This study aims to investigate the diversity in reproductive and morphological traits that can be useful in selective breeding programs for improving colony performance and survivability. The studied animals came from a population bred by a professional queen breeder in Northern Italy. Heritability and genetic correlations were estimated. According to our results, some of the traits showed good variability and they could be included as breeding goals in selection programs. Improving the quality of queens could directly impact honeybee colonies’ performance and survivability. Ultimately, it represents an added value to a queen bee-breeder company.

**Abstract:**

The quality of the honeybee queen has an important effect on a colony’s development, productivity, and survival. Queen failure or loss is considered a leading cause for colonies’ mortality worldwide. The queen’s quality, resulting from her genetic background, developmental conditions, mating success, and environment, can be assessed by some morphological measures. The study aims to investigate variability for traits that could assess the quality of the queen. Related animals were enrolled in this study. Variance components were estimated fitting a mixed animal model to collected data. Heritabilities of body and tagmata weights ranged from 0.46 to 0.54, whereas lower estimates were found for the tagmata width and wing length. Heritabilities estimated for the spermatheca diameter and volume, number of ovarioles, and number of sperms were 0.17, 0.88, 0.70, and 0.57, respectively. Many phenotypic correlations related to size were high and positive, while weak correlations were found between morphology and reproductive traits. Introducing a queen’s traits in a selection program could improve colonies’ survivability. Further research should focus on better defining the correlations between the individual qualities of a queen and her colony’s performance.

## 1. Introduction

Honey bees (*Apis mellifera*) are social insects who live in colonies characterized by a cooperative system of brood care, overlapping generations, and reproductive division of tasks [1]. In such an organized bio-social structure, the queen is the fertile female whose main duty is to lay eggs [2]. Moreover, the queen maintains the colony cohesion through a continuous production of a bouquet of pheromones which are actively spread within the nest. They prevent the workers from substituting the queen and developing their ovaries [3,4,5,6]. The colony development, productivity, and survival depend substantially on the health and fitness of its queen and the drones which she mated with [7,8,9,10]. A bee colony is adversely affected if the queen shows any defects or becomes ill and ceases to lay eggs [11,12,13,14]. Besides agrochemicals, parasites, and pathogens, failure or loss of the queen have been considered the most important factors leading to colony losses worldwide, especially when it occurs outside the natural queen rearing season [15,16,17,18]. A poor quality queen is a factor that consistently ranks among the top reasons for bee colony failure [18,19].

There are many measures that can be correlated to queen “quality”, which results from her genetic background, her developmental conditions, mating success, and adult environment including the beekeeper’s management [20,21,22]. The most intuitive are physical measures of the queen, such as the body weight, which was found to be significantly correlated with her fitness and colony productivity [23,24,25,26,27,28,29,30]. Weight was also found to be positively associated with higher acceptance of queens in new colonies [30,31,32,33]. Bodyweight was also positively correlated with reproductive organs of the queen such as ovaries and number of ovarioles, the diameter of the spermatheca, and the number of stored spermatozoa [23,24,27,28,34]. Amiri et al. [35] concluded in their review that the body weight of a queen could represent an integrative measure of the size and physiological condition. Therefore, it could be considered one of the most informative indicators of the queen’s quality. Researchers investigated any possible association between weight and reproductive organs [24,26,34,36]. In Delaney et al. [24], thorax width was found positively correlated with the number of stored sperm and mating frequency. Meanwhile, other studies reported no correlation between thorax width and ovarioles number, ovary weight, or mating number [26,34,36].

In a mated and egg-laying queen, the ovaries are the organs involved in the production of eggs. They are present in couples and occupy most of the abdominal cavity [2]. They consist of a bundle of ovarioles (ca. 150 each), which are long tubules containing egg cells, nurse cells, and follicle cells [37]. Ovary development takes place soon after the mating flights and it is associated with distinct gene-expression patterns in the brain and ovaries, and physiological and behavioral changes in the queen [38,39,40]. The weight of the ovaries in a mature egg-laying queen not only depends on the number of ovarioles but also on the number and developmental stage of eggs they contain [35]. The ovaries’ weight was reported as one of the internal physical criteria to assess the reproductive potential of honey bee queens [24,41,42]. Ovary dimensions and fertility are reported to be positively correlated [43]. The number of ovarioles can be evaluated at any time during the life of a queen [37]. Queen size, ovary size, and symmetry are affected by larval nutrition [27,44]. If the queen is artificially reared, the age of the grafted larvae is critical and also influences natural queen supersedure [27,43,44,45].

Besides the ovaries, the queen’s reproductive system includes one spermatheca. The spermatheca is a small spherical shaped organ that preserves living sperms after mating for a lifelong period of time [2]. The spermatheca’s size is another measure of internal physical queen quality, under the assumption that a larger spermatheca could hold a larger volume of semen [35]. Its size can be measured with or without the tracheal nets, and its diameter should be larger than 1.2 mm for high-quality queens [25,37]. This measurement was used as a direct estimation of the volume and as an indirect estimation of the theoretical maximum number of spermatozoa stored in spermatheca [26,28,34,37]. The size of the spermatheca is influenced by rearing conditions and genetics, and it is inversely proportional to the larval age at which the queen was reared from [26,43]. Queens raised from newly hatched larvae showed larger spermatheca [42,43,45]. However, the spermatheca is rarely filled completely, as the semen’s occupied volume in experimental queens was reported to be on average 47% [26,34].

From a hypothetical perspective, a “high quality” queen should therefore be morphologically defects-free, and it should have a large body, spermatheca, and ovaries in order to store a high number of spermatozoa and lay a copious number of eggs, preferably over 2000 eggs per day [14,46].

In this study, both external and internal physical queen traits were investigated. Such traits include: body weight, weight and width of the tagmata (head, thorax, and abdomen), length of the right forewing, diameter and volume of the spermatheca, number of sperms in the spermatheca, and number of ovarioles. Ovarioles were counted instead of weighed since the ovary’s weight could be influenced by the developmental stages of the eggs they contain, as pointed out by Amiri et al. [35].

The aim of this research was to investigate phenotypic and genetic variability of the above-mentioned traits for queen quality in a small population bred by a professional queen breeder in Northern Italy.

## 2. Materials and Methods

The queens were provided by an Italian queen-breeding and beekeeping company that produces and sells about 400–600 queens per week, from spring to late summer. The rearing of the queens was characterized by a standardized production system and by traceability of both maternal and paternal lines of each queen (the pedigree). The standardized rearing system consists of using only queen-less finisher colonies. These colonies are fed and treated in a standardized way to provide uniform quality. Specifically, these colonies receive new brood from one single apiary on a regular basis. The “brood-donor” apiary is composed of genetically uniform colonies. The grafts are inserted in the finisher colonies for a week. Afterwards, the royal cells are collected and incubated at 34.5 °C for 11 days. After incubation, the cells are brought to the mating station where they are inserted in the mating nuclei. This process was carried out by the same operators following a strict timetable along the season, which minimizes any potential error variance due to management practices.

This study was conducted on 147 queens bred during spring/summer seasons of 2017 (*n* = 70) and 2018 (*n* = 77). The analyzed queens were bred at different times of the production season reported as the ordinal number of the week of the year in which the mated queen was harvested from the mating nucleus. All queens naturally mated in the same area within the year. The queens were bred in groups of sisters from 10 maternal lines in 2017, and 7 maternal lines in 2018. Furthermore, the maternal lines shared common ancestors. The maternal lines queens mated at an isolated mating station where each year a group of 12–15 drone-producing colonies provided drones. In this way, a pedigree could also be traced from the paternal side.

### 2.1. Animal Sampling and Transport

**Figure 1:**
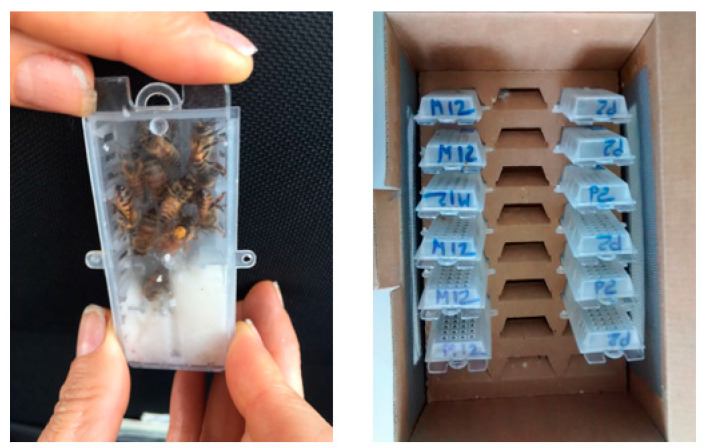
**Left**: queen transport cage; **Right**: box for queen transport.

The newly mated queens were harvested by the operators after assessing that each queen was successfully mated at the mating station. This was accomplished by checking the presence of viable female brood in the mating nucleus. The queens were shipped to the laboratory in suitable queen cages with feed and a sufficient number of attending worker bees (Figure 1, left). During transport, they were kept in a cardboard box with spacer support for the cages and holes for aeration (Figure 1, right). This transport system is one of the most common ways to transport or ship living bees in Italy.

### 2.2. Freeze Immobilization

For easier handling during the first inspection, the queens were stored in the freezer at −20 °C for 15–17 min. The cold anesthetized the animals and induced them into an immobile state.

### 2.3. Morphological Measures

After cold immobilization, the first analysis was to evaluate the exterior state of the insect for the detection of any macroscopic defects e.g., missing legs, wings, antennae, or any visible injury of the body. These assessments were carried out under a stereomicroscope. Afterwards, queens were euthanized by decapitation and processed. The way morphological traits were measured differed between 2017 and 2018.

**Figure 2:**
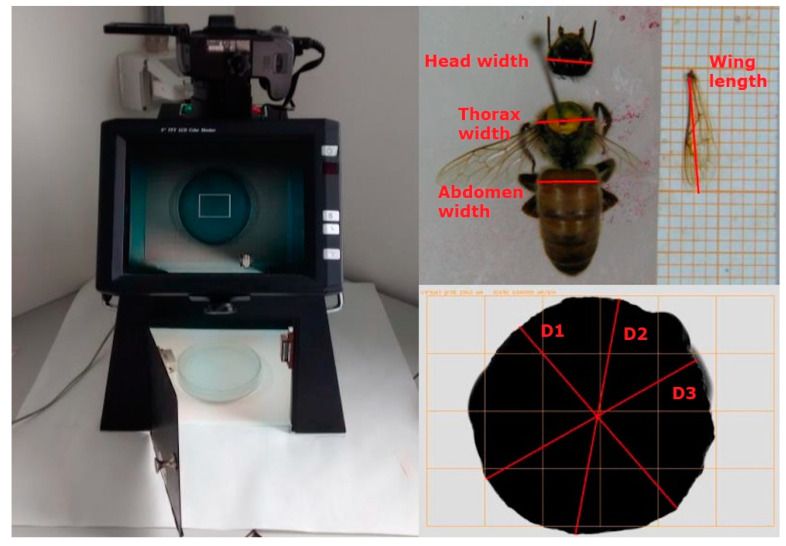
Left: image acquisition set-up; top right: example of queen tagmata widths for morphological measures; bottom right: example of the three spermatheca diameter measures (D1 = diameter 1; D2 = diameter 2; D3 = diameter 3).

In 2017, after the sacrifice, the insect was pinned onto a dissection dish previously filled with paraffin in order to form a basal layer to permit the fixing of the animal on the paraffin surface with entomological needles. The right forewing was detached from the body and laid beside the insect. The dish with the insect was numbered and photographed for post hoc morphological measurements with ImageJ software (U.S. National Institutes of Health, Bethesda, MD, USA) [47]. Every image contained a reference scale (graph paper) to determine the pixel/mm ratio. After the acquisition of the image, every queen was weighed on an analytical scale recording the weight of the entire insect body (bw), and separately also the weight of the head (hw), abdomen (aw), and thorax (tw). Other metrics were recorded with image analysis as follows: the head width (hwi) was assessed measuring the distance between the two compound eyes in their widest frontal part; the thorax width (twi) was recorded measuring the distance between the two tegulae, which are the scales on the mesothorax that overlaps the root of the forewing; the abdomen width (awi) was assessed measuring the width of the first apparent abdominal tergite in its wider part. Finally, the length of the right forewing (wl) was measured from the humeral plate to the apex. In 2018, the above-described measurements were taken manually using a digital caliper right after the sacrifice of the insect. Figure 2 shows how measurements were taken both with image analysis and manually.

### 2.4. Abdomen Dissection

**Figure 3:**
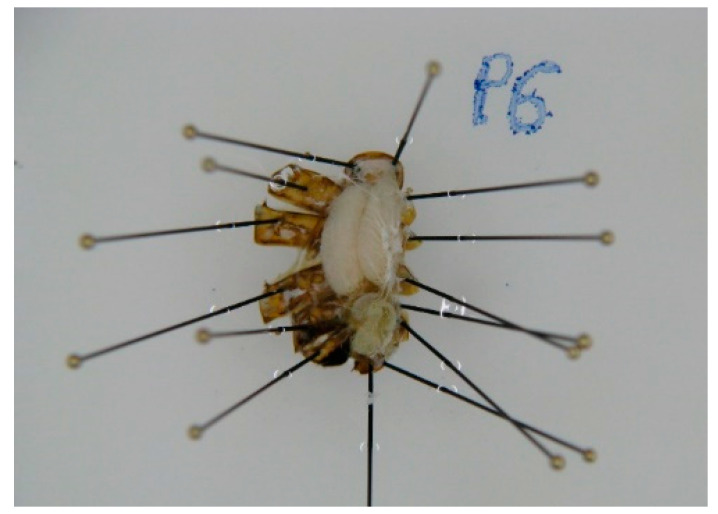
Queen abdominal cavity after dissection (P6 in the figure was a note for tracking the sample identity).

In order to assess the reproductive characteristics of the queen, the abdomen was dissected following the methods described in Porporato et al. [46]. The abdomen was kept in a ventral position by two proximal needles and one distal needle. Each abdomen was dissected cutting the junction between dorsal and ventral tergites with a scalpel and pulling away one by one the dorsal tergites with tweezers and fixing them beside it with needles (Figure 3). As soon as the abdominal cavity was opened, the abdomen was submerged in physiological solution (NaCl 0.9%) to prevent the drying out of the internal organs.

### 2.5. Spermatheca Extraction and Analysis

As soon as the abdomen was opened, the spermatheca was extracted and put on a glass slide. In 2017, the spermatheca was photographed on a slide under the microscope, and its diameter (ds) was measured with ImageJ software. In 2018, the diameters were measured by hand with a digital caliper. Since the spermatheca of a queen does not have a perfect spherical shape, the diameter was calculated as the average of three measurements. The volume of the spermatheca (vs) was calculated as the volume of a sphere.

To assess the concentration of semen, the spermatheca was popped in 1 mL of physiological solution (NaCl 0.9%). The concentration of sperm (sp) was assessed using a standard haemocytometer chamber (Burker Camera) under a light microscope [48].

### 2.6. Ovarioles Count

**Figure 4:**
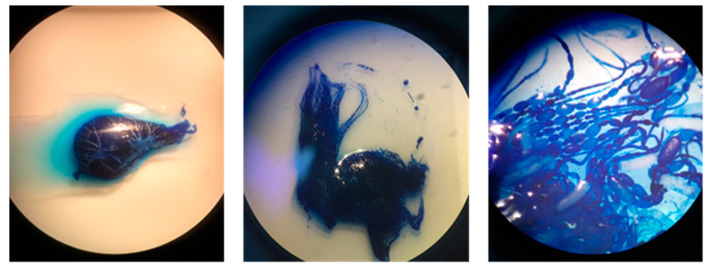
Ovarioles count. From left to right: an ovary after staining, an ovary during ovarioles count, detail of ovarioles’ structure.

Only the ovarioles of the right ovary were counted [37]. The number of ovarioles (o) was estimated with a method derived from Porporato et al. [46]. The right ovary was removed from the abdominal cavity and kept in staining solution for ca. 10 min (methylene Blue Trihydrate, 0.5%, Sigma-Aldrich, St. Luis, MO, USA). After dying, the ovary was washed with physiological solution, placed on a slide, and analyzed under a stereomicroscope. The ovarioles were separated with the help of a dissecting needle and counted one by one, without cutting the ovary (Figure 4).

### 2.7. Statistical Analysis

For the analysis, estimates of the genetic relationships among the studied queens are required. The methods of Brascamp and Bijma [49] were used to estimate relationships assuming the queens mated with 12 drones. The pedigree file was built following the procedure described in Brascamp et al. [50]. To estimate heritability and genetic correlations, the statistical package ASReml and the pin function of the nadiv package were used in the computing environment R [51,52,53].

First, a univariate mixed animal model [54] for each trait was fitted, using the following model:

(1) $$y_{ijk}=\mu +wy_{i}+\alpha_{j}+\varepsilon_{ijk},$$

where μ is the overall mean of the trait, $wy_{i}$ represents the fixed effect of the *i*th combination of the week of the year in which the queen was harvested (*i* = 1, 15, specifically 6 weeks in 2017, 9 weeks in 2018), $\alpha_{j}$ represents the random genetic effect of the *j*th queen (*j =* 1147), $\varepsilon_{i,j,k}$ represents the random error term of the *k*th observation. This model allowed us to estimate the heritability of each measured phenotype.

Secondly, a bivariate approach was used for each combination of traits fitting the same model above described. The bivariate analysis allowed us to estimate both phenotypic and genetic correlations between the measured traits.

## 3. Results and Discussion

### 3.1. Defects and Descriptive Statistics

Every analyzed queen was free from macroscopic external defects. However, some internal defects were observed. The most frequent defect observed in the 16% of the queens was an abnormal intestinal tract, which appeared swollen and brownish/yellowish in color; in the 9% melanosis of the ovary was observed; in the 8% of queens enteroliths were observed, which are small stones in the intestinal tract also described in Porporato et al. [46]; in 2.7% atrophy of at least one ovary was observed. Finally, less than 1% of cases showed empty or dark-colored spermatheca. Potential causes for the aforementioned defects were not investigated further. It can be assumed they arose from either abiotic factors (transport conditions, handling at harvest) or from biotic factors (pathogens). 

Mean, standard deviation (SD), and coefficient of variation (CV) of recorded traits are presented in Table 1.

The number of sperm and volume of spermatheca were very variable, as their CV resulted 82.8% and 41.7%, respectively. Other measures such as tagmata weights, diameter of spermatheca, and number of ovarioles were less variable and their CV ranged from 10.1% to 17.9%. Concerning body weight, our result was in good agreement with values reported by the literature [24,25,26,46,55]. Records from Italian studies were of 186 ± 24 mg average body weight reported by Porporato et al. [46] and 221 ± 3.09 mg reported by Hatjina et al. [25]. Head and thorax widths were in agreement with Tarpy et al. [26], Delaney et al. [24], and Hatch et al. [45]. While no references are currently available on abdomen dimensions, the average width of the first apparent tergite was 4.8 ± 0.21 mm. Furthermore, queen tagmata weights were not found in the literature. Our data suggest that the abdomen’s weight was the most variable among the three tagmata. It could be argued that the eggs’ developmental stages may differ along each ovariole and the nutritional state of the queen at the moment of the analysis (filling of the intestinal tract) might be the source of such variation. The length of the right forewing was in agreement with previous reports [24,55]. Concerning the reproductive organs of the queen, our result on the number of ovarioles was in range considering the 74 ± 14 reported by Porporato et al. [46] and the 174 reported by Hatijna et al. [25]. Results of spermatheca size and number of sperm in the spermatheca were in agreement with the majority of previously reported studies [24,25,26,46].

### 3.2. Heritabilities, Genetic and Phenotypic Correlations

Genetic parameters are reported in Table 2; heritabilities were estimated with univariate analyses of the traits, while genetic and phenotypic correlation were estimated with a bivariate approach.

Heritabilities ranged from 0.17 to 0.88 with rather high standard errors which might be explained by the sample size. In particular, for body weight and for individual tagmata weight, heritabilities resulted to be in a narrower range between 0.46 and 0.54. On the other hand, for tagmata width, the higher estimate was found for thorax width (0.42 ± 0.32), followed by the head width (0.26 ± 0.27) and the lowest was the estimate for the abdomen width (0.13 ± 0.26). The estimate for the length of the right forewing was 0.30 ± 0.29. Estimates for diameter and volume of the spermatheca resulted in 0.17 ± 0.34 and 0.88 ± 0.39 respectively. The increase of sv heritability compared to sd heritability could be explained by the way sv was calculated from ds. Indeed, sv was estimated from sd applying the formula of the volume of the sphere. This transformation increased the total phenotypic variance of trait sv by a coefficient (4/3 × π), without affecting the sv error variance. Therefore, the higher the genetic variance, the higher the heritability of sv trait. This result should be verified as the volume of spermatheca was approximated to that of a sphere, although this organ may show different shapes [46]. The estimates for the number of ovarioles and for the number of sperm in the spermatheca were 0.70 ± 0.35 and 0.57 ± 0.35, respectively. There are no results in the literature so far on the heritabilities for the traits measured in this study. Overall, the results show considerable genetic variability in the studied population.

Concerning morphological traits (bw, hw, tw, aw, hwi, twi, awi, wl), phenotypic correlations ranged from 0.16 to 0.88. Highly correlated traits were body weight with abdomen weight (0.88 ± 0.03) and thorax weight (0.67 ± 0.06); moreover, a correlation of 0.80 ± 0.04 resulted between abdomen and thorax widths. Lower correlations were observed between morphological traits and the length of the right forewing. We observed very low or close to zero phenotypic correlations among reproductive traits (ds, vs., o, sp) except for the correlation between sd and sv (0.97 ± 0.01). The latter is likely explained by the fact that sv is derived by sd using the formula to estimate the volume of a sphere. Remarkably, according to our results, reproductive traits do not seem to be associated with morphological measures. Our results are in agreement with Corbella and Gonçalves [56], Hatch et al. [45], and Jackson et al. [36] who also reported the lack of phenotypic correlation between the body weight of a queen and the number of ovarioles. In addition, no phenotypic correlation between the number of sperms and spermatheca diameter was found, as previously reported by Jackson et al. [36].

As expected, our genetic correlations estimates were affected by large standard errors due to the limited number of individuals in our dataset. Therefore, estimates should be considered with caution. Among morphological traits, tagmata weights showed high genetic correlations which ranged from 0.61 to 0.99. High and positive genetic correlations were found between the weight and width of the head (0.97 ± 0.46) and thorax (0.83 ± 0.31), respectively. There was also a high genetic correlation between thorax width and abdomen width (0.74 ± 0.34). In addition, high genetic correlations were observed between wl and hw (0.92 ± 0.71), tw (0.74 ± 0.38), hwi (0.96 ± 0.48), and twi (0.98 ± 0.31). The high relationship between lw and thorax dimensions may be explained by the fact that wings grow on the thorax, the locomotive tagma which groups the legs and the wings. It could be argued that during the insect’s development they are tightly and jointly regulated. The dimension of the spermatheca positively correlated with tw (0.78 ± 0.39) and hwi (0.89 ± 0.48). As expected, the diameter and volume of the spermatheca correlation were near to one (0.99 ± 0.02). Positive genetic correlations were found between wing length and number of ovarioles (0.79 ± 0.59) and number of sperms (0.57 ± 0.52). The association between wing length and these reproductive traits may be explained by the paramount function of the wings during the mating flight, which depends on the wings’ movements. The very high negative genetic correlation between the dimension of the spermatheca and the number of sperms it contains was surprising (−0.96 ± 0.72 with ds, −0.70 ± 0.56 with vs), previous reports showed that the spermatheca is often not totally filled after mating [26,34].

## 4. Conclusions

Beekeepers have long selected queens, choosing the “best” on the basis of phenotypic desired features, mainly body size and color. The purpose set at the beginning of this study was to investigate a series of traits that could be useful for the evaluation and the selection of any qualities of a queen. Statistical analysis confirmed the existence of certain rather intuitive correlations between some morphological measures. Other results shed light on aspects that were counterintuitive in principle, i.e., the absence of phenotypic correlation between morphological measures and reproductive traits. Heritability is a parameter that generally describes how easily parents transmit to their offspring a certain phenotype and which has, on the other hand, a practical operational utility for breeders and selection programs. The heritability of body weight, spermatheca volume, number of ovarioles, and number of stored sperm look like promising breeding goals, given the observed genetic variability. It would be interesting to reproduce and extend this study to other traits, and to a larger number of individuals (also considering different breeds), to confirm or update the conclusions from this study. Moreover, it would be pivotal to extend such studies to colonies’ performances. In fact, phenotypic or genetic correlations between colony performance and queen quality, estimated with the animal model, are completely lacking in the literature.

In conclusion, monitoring and introducing queen’s traits as breeding goals in a genetic improvement program represent an appealing plan of approach to the overall decline of queens reported in recent years from both the beekeepers and the scientific community. Improving the overall quality and reproductive traits of queens could directly impact colonies’ performance and survivability. Ultimately, it represents an added value to a queen bee-breeder company.

## Figures and Tables

**Table 1:** List of traits measured on queens with abbreviations used in the text, unit of measure, number of observation (N), mean, standard deviation (SD), and coefficient of variation (CV).

Trait	Abbreviation	Unit	N	Mean	SD	CV (%)
Body weight	bw	mg	147	195.90	19.84	10.1
Head weight	hw	mg	147	13.22	1.83	13.8
Thorax weight	tw	mg	147	77.93	8.17	10.5
Abdomen weight	aw	mg	147	104.76	15.28	14.6
Head width	hwi	mm	147	3.67	0.18	4.9
Thorax width	twi	mm	147	4.67	0.26	5.6
Abdomen width	awi	mm	147	4.80	0.21	4.4
Wing length	wl	mm	147	10.07	0.62	6.2
Spermatheca diameter	sd	mm	147	1.32	0.18	13.6
Spermatheca volume	sv	µl	147	1.27	0.53	41.7
Number of Ovarioles	o	n	147	141	25	17.9
Number of Sperms	sp	n million	143	3.6	2.9	82.8

**Table 2:** Heritabilities (diagonal and in bold), genetic (above diagonal) and phenotypic (below diagonal) correlations estimates for traits measured on queens. Standard errors for heritability estimate are reported in brackets.

Trait	Body Weight	Head Weight	Thorax Weight	Abdomen Weight	Head Width	Thorax Width	Abdomen Width	Wing Length	Diameter Spermatheca	Volume Spermatheca	Ovarioles Number	Sperm Count
Body weight	**0.54** **(0.34)**	0.80 (0.31)	0.92 (0.30)	0.84 (0.17)	0.47 (0.50)	0.34 (0.50)	−0.22 (1.10)	0.17 (0.62)	0.23 (0.76)	0.40 (0.40)	−0.13 (0.50)	−0.13 (0.52)
Head weight	0.39 (0.10)	**0.51** **(0.35)**	0.99 (0.36)	0.61 (0.45)	0.97 (0.46)	0.34 (0.53)	−0.56 (1.05)	0.92 (0.71)	−0.44 (0.68)	−0.44 (0.50)	0.28 (0.50)	0.23 (0.57)
Thorax weight	0.67 (0.06)	0.19 (0.11)	**0.50** **(0.39)**	0.98 (0.69)	−0.01 (0.79)	0.83 (0.31)	0.54 (0.75)	0.74 (0.38)	0.78 (0.39)	0.61 (0.41)	−0.18 (0.53)	0.15 (0.58)
Abdomen weight	0.88 (0.03)	0.31 (0.10)	0.29 (0.10)	**0.46** **(0.34)**	0.47 (0.54)	−0.14 (0.68)	−0.60 (1.19)	−0.45 (0.71)	−0.24 (1.05)	0.06 (0.54)	−0.15 (0.53)	−0.3 (0.52)
Head width	0.36 (0.09)	0.31 (0.10)	0.28 (0.10)	0.27 (0.10)	**0.26** **(0.27)**	0.21 (0.67)	0.15 (1.08)	0.96 (0.48)	0.89 (0.48)	0.7 (0.52)	0.55 (0.58)	0.63 (0.56)
Thorax width	0.34 (0.10)	0.30 (0.10)	0.28 (0.11)	0.26 (0.10)	0.32 (0.09)	**0.42** **(0.32)**	0.74 (0.36)	0.98 (0.31)	0.59 (0.57)	0.44 (0.43)	0.02 (0.53)	−0.17 (0.56)
Abdomen width	0.39 (0.09)	0.17 (0.10)	0.26 (0.10)	0.33 (0.10)	0.25 (0.09)	0.80 (0.04)	**0.13** **(0.26)**	0.33 (0.90)	−0.22 (2.07)	0.44 (0.65)	−0.59 (0.89)	0.26 (0.96)
Wing length	0.34 (0.10)	0.16 (0.10)	0.36 (0.10)	0.20 (0.11)	0.26 (0.10)	0.39 (0.09)	0.30 (0.09)	**0.30** **(0.29)**	0.43 (0.81)	0.40 (0.50)	0.79 (0.59)	0.57 (0.52)
Diameter spermatheca	0.21 (0.10)	0.04 (0.12)	0.18 (0.12)	0.20 (0.10)	0.08 (0.12)	0.15 (0.11)	0.13 (0.10)	0.10 (0.10)	**0.17** **(0.34)**	0.99 (0.02)	−0.42 (0.64)	−0.96 (0.72)
Volume spermatheca	0.22 (0.13)	0.03 (0.13)	0.11 (0.12)	0.18 (0.13)	0.02 (0.12)	0.16 (0.13)	0.16 (0.12)	0.09 (0.12)	0.97 (0.01)	**0.88** **(0.39)**	−0.31 (0.42)	−0.70 (0.56)
Ovarioles number	0.01 (0.12)	0.15 (0.12)	−0.003 (0.12)	−0.02 (0.12)	0.08 (0.11)	0.04 (0.12)	0.03 (0.12)	0.02 (0.12)	−0.01 (0.12)	−0.05 (0.13)	**0.70** **(0.35)**	0.08 (0.48)
Sperm count	0.03 (0.12)	0.03 (0.12)	0.09 (0.12)	−0.01 (0.12)	0.10 (0.11)	0.04 (0.12)	0.08 (0.11)	0.17 (0.11)	−0.07 (0.12)	−0.07 (0.13)	0.05 (0.12)	**0.57** **(0.35)**

## Data Availability

The data presented in this study are available on request from the corresponding author.
